# Phosphodiesterase activity is regulated by CC2D1A that is implicated in non-syndromic intellectual disability

**DOI:** 10.1186/1478-811X-11-47

**Published:** 2013-07-04

**Authors:** Azza Al-Tawashi, Chris Gehring

**Affiliations:** 1 Center for Molecular Discovery, Verna and Marrs McLean Department of Biochemistry and Molecular Biology, Baylor College of Medicine, 77030 Houston, TX, USA; 2 Division of Chemical and Life Sciences and Engineering, King Abdullah University of Science and Technology, 23955 Thuwal, Saudi Arabia

**Keywords:** Phosphodiesterase activity, cAMP-PKA pathway, CC2D1A, Non-syndromic intellectual disability

## Abstract

**Background:**

Cyclic adenosine 3^′^,5^′^-monophosphate (cAMP) is a key regulator of many cellular processes, including in the neuronal system, and its activity is tuned by Phosphodiesterase (PDE) activation. Further, the CC2D1A protein, consisting of N-Terminal containing four DM14 domains and C-terminal containing C2 domain, was shown to regulate the cAMP-PKA pathway. A human deletion mutation lacking the fourth DM14 and the adjacent C2 domain results in Non Syndromic Intellectual Disability (NSID) also referred to as Non Syndromic Mental Retardation (NSMR).

**Findings:**

Here we demonstrate that in Mouse Embryonic Fibroblasts (MEF) CC2D1A co-localizes with PDE4D in the cytosol before cAMP stimulation and on the periphery after stimulation, and that the movement to the periphery requires the full-length CC2D1A. In *CC2D1A* mouse mutant cells, the absence of three of the four DM14 domains abolishes migration of the complex to the periphery and causes constitutive phosphorylation of PDE4D Serine 126 (S^126^) via the cAMP-dependent protein kinase A (PKA) resulting in PDE4D hyperactivity. Suppressing PDE4D activity with Rolipram in turn restores the down-stream phosphorylation of the “cAMP response element-binding protein” (CREB) that is defective in mouse mutant cells.

**Conclusion:**

Our findings suggest that CC2D1A is a novel regulator of PDE4D. CC2D1A interacts directly with PDE4D regulating its activity and thereby fine-tuning cAMP-dependent downstream signaling. Based on our *in vitro* evidence we propose a model which links CC2D1A structure and function to cAMP homeostasis thereby affecting CREB phosphorylation. We speculate that CC2D1A and/or PDE4D may be promising targets for therapeutic interventions in many disorders with impaired PDE4D function such as NSID.

## Background

Cyclic AMP regulates a host of cellular functions that include the activation of cAMP-dependent protein kinase A (PKA) that in turn regulates processes such as transcription, cell growth and differentiation, metabolism and ion channel conductivity [1,2]. Therefore, alterations in cellular cAMP homeostasis, regulated by adenylate cyclases (ACs) and phosphodiesterases (PDEs) are likely to profoundly affect these cellular processes [3]. The resting concentration of cAMP inside mammalian cells is about 10^-7^ M and extracellular signals can cause cAMP levels to change by > 20 fold in seconds which can diffuse rapidly (130 to 700 μm^2^ sec^–1^) [4]. In animal cells, cAMP can exert its effects via PKA that consists of a tetrameric holoenzyme composed by two regulatory subunits constitutively linked to two catalytic subunits [5]. Upon stimulation cAMP binds to the regulatory subunits, causing a conformational change which results in the release of catalytic subunits. Once separated, catalytic subunits become activated and catalyze the phosphorylation of specific serines or threonines of target proteins [6]. Activated PKA catalytic subunits (Cs) translocate to the nucleus to phosphorylate the cyclic AMP response element (CREB) at Serine 133 (S^133^) initiating the transcription of target genes [7-10]. Compartmentalization of the cAMP active pool occurs through the cAMP module binding to A Kinase Anchoring Proteins (AKAPs) [11]. Tethering the PKA regulatory subunit(R) to specific subcellular sites by binding AKAPs helps to assure specific phosphorylation of specific targets and thus prevents uncontrolled phosphorylation. PDEs in turn reduce cAMP concentration thereby tuning the signal down or turning it “off” [12]. A number of distinct isotypes of PDEs modulate the amplitude, length and subcellular distribution of the cAMP signal [13]. Intracellular localization of PDEs also contributes to compartmentalization of cyclic nucleotide signaling [14]. Activation of PKA is tuned by the activity of PDE4D that decreases cAMP levels at the site where PKA is located while PKA phosphorylation of PDE4D is important to fully activate PDE4 enzymes [15,16]. This balanced system enables discrete signaling.

Recently, CC2D1A has been shown to be a novel regulator of the cAMP-PKA pathway [17]. Amino acid sequence analyses of the Coiled-coil and C2 domain-containing 1A (CC2D1A) suggested that it consists of a C2 calcium-dependent phospholipid-binding domain, and four conserved *Drosophila melanogaster* 14 (DM14) domains specific to this protein family with uncharacterized function(s) [18]. Mutant mice with a truncated CC2D1A show defective cAMP-PKA activation and CREB (S^133^) phosphorylation [17].

Interestingly, in NSID patients, the CC2D1A mutant protein has only the first three of the four DM14 domains and carriers have no physical defects but are intellectually disabled [19,20], while the mouse mutant CC2D1A has only a single intact DM14 domain causing death eight to twelve hours after birth, pointing to an essential role of the second and third DM14 domains. Here we set out to characterize the role of CC2D1A during cAMP-dependent stimulation and suggest that its specific function may make a promising drug target.

## Results and discussion

### PDE4D co-localizes with CC2D1A before and after cAMP signaling stimulation

**Figure 1:**
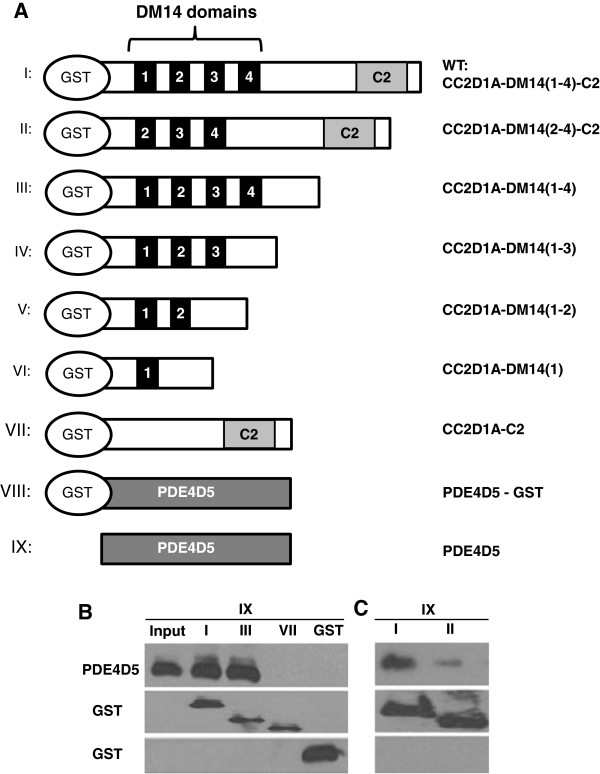
***In vitro***-**binding of CC2D1A to PDE4D5. A**. Schematic diagram of GST-CC2D1A and GST-PDE4D5 fusion constructs. **B**. Western blot of *in vitro* binding assays of recombinant proteins CC2D1A (fragments I, III, VII and GST) and recombinant PDE4D5 (fragment IX) probed with anti-PDE4D (upper panel) or anti-GST (middle panel and lower panel). The “Input” was purified recombinant PDE4D5 (fragment IX). **C**. Western blot of *in vitro* binding assays of recombinant proteins CC2D1A (fragments I and II) and recombinant PDE4D5 (fragment IX) probed with anti-PDE4D (upper panel) or anti-GST (middle panel and lower panel).

CC2D1A was previously shown to associate with PDE4D5 even in the *CC2D1A* mutant cells and in brain tissue [17]. In order to characterize CC2D1A interactions with PDE4D5, a series of *in vitro* pull-down experiments were performed (Figure 1). The different recombinant GST-tagged CC2D1A proteins (fragments I, II, III, and VII) (Figure 1A) were immobilized on glutathione beads and incubated with purified PDE4D5 (IX) (Figure 1A) and PDE4D5-binding was assessed by western blot. PDE4D5 binds to full-length CC2D1A (I) and the CC2D1A (III) fragments, but not to the CC2D1A (VII) fragment suggesting that CC2D1A DM14 domains are essential for binding PDE4D5 (Figure 1B). In addition, CC2D1A-PDE4D5 binding was almost completely abolished in the absence of the first DM14 domain (fragment II) (Figure 1C). This is consistent with previously reported observations that PDE4D5 can be immunoprecipitated with the mouse CC2D1A mutant form that contains only the first DM14 domain [17], a construct that is similar to fragment VI. We therefore conclude, firstly, that CC2D1A binds PDE4D5 directly and that this binding occurs on the N-terminus and within the DM14 domains and secondly, that the first DM14 domain is essential for the binding. Thirdly, the C2 domain is not required for binding.

**Figure 2:**
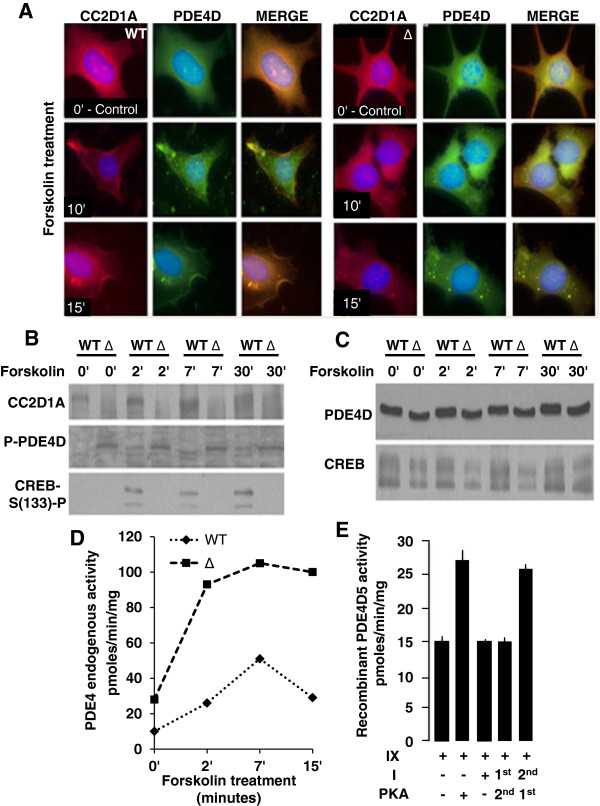
**CC2D1A regulates PDE4D activity. A**. Immunocytochemistry of forskolin induction time course 0, 10 and 15 minutes of wt and CC2D1A mutant (Δ) Mouse embryonic fibroblasts (MEF) co-stained with anti-CC2D1A and anti-PDE4D. The mouse mutant form (Δ) is similar to fragment VI since it contains only the first DM14 domain. **B**. Western blot of forskolin induction time course 0, 2, 7 and 30 minutes of wt and CC2D1A mutant (Δ) MEF cells probed with anti-CC2D1A (upper panel), anti- phospho PDE4D (middle panel) or anti-phospho CREB (lower panel)(n = 7). **C**. Western blot of forskolin induction time course 0, 2, 7 and 30 minutes of wt and CC2D1A mutant (Δ) MEF (shown in **B**) cells probed with anti-PDE4D (upper panel) or anti-CREB (lower panel). D. Endogenous PDE4 activity in forskolin stimulated wt and CC2D1A mutant (Δ) MEF cells. **E**. *In vitro* recombinant basal PDE4D5 (fragment IX) activity in the presence and absence of PKA and/or CC2D1A (I).

Given that firstly, CC2D1A migrates to the cell periphery after cAMP-stimulation [17] and, *in vitro* binding of CC2D1A to PDE4D5 (Figure 1), we tested if PDE4D co-localizes with CC2D1A at the periphery. To test this we stimulated wild type (wt) and *CC2D1A* mutant Mouse Embryonic Fibroblast (MEF) cells with forskolin, fixed them and co-stained them with anti-CC2D1A and anti-PDE4D antibodies. The results show that PDE4D and CC2D1A co-localize in the cytosol prior to stimulation and accumulate at the cell periphery after stimulation (Figure 2A). Additionally, although the CC2D1A - PDE4D co-localization in the cytosol was observed in the *CC2D1A* mutant cells before stimulation, accumulation at periphery does not occur after stimulation indicating the importance of CC2D1A and PDE4D binding in PDE4D accumulation at the periphery (Figure 2A).

### The CC2D1A-PDE4D binding regulates PDE4D activity

**Figure 3:**
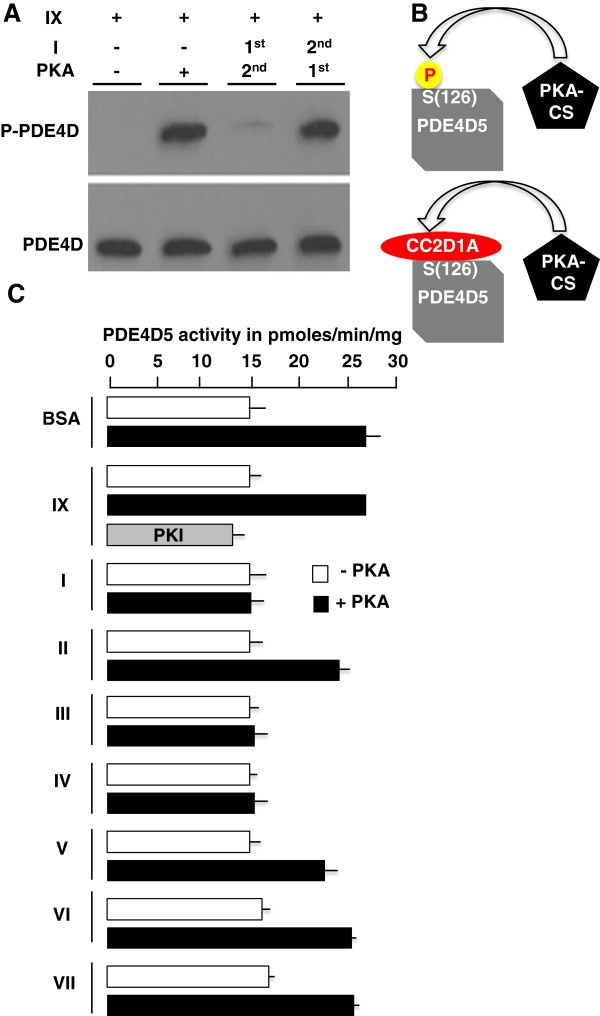
**Mapping of the function of DM14 domains. A**. Western blot of recombinant PDE4D5 protein (fragment IX) probed with anti-phospho PDE4D or anti-PDE4D in the presence and absence of PKA or CC2D1A (I). **B** schematic diagram of PDE4D phosphorylation at (S^126^) by PKA in the presence and absence of CC2D1A. **C**. *In vitro* PDE4D activity assay of recombinant PDE4D5 (IX) after pre-incubation with BSA, PKI (specific PKA inhibitor) or CC2D1A (fragments I, II, III, IV, V, VI, VII) in the presence and absence of PKA. BSA was used as a control to validate the specificity of the responses of the different CC2D1A constructs on the inhibition of PDE4D5 activity in the presence of PKA.

Since PKA phosphorylation of PDE4D (S^126^) causes activation [21], we investigated whether PDE4D phosphorylation was affected in *CC2D1A* mutant MEF cells. When cells were stimulated with forskolin, lysed and western blotting was performed using anti-phospho-PDE4D and anti-PDE4D antibodies, we noted that the level of PDE4D phosphorylation was consistently increased in the mutant (n = 7) suggesting that PDE4D may be more active in the mutant even before stimulation which corresponds with CREB phosphorylation defect in the *CC2D1A* mutant cells on the same western blot (Figure 2B). To validate the sample loading and the phospho-PDE4D and phospho-CREB bands, we re-stained the same blot with anti-PDE4D and anti-CREB (Figure 2C). Given that PDE4 activity increases by 2–3 fold after PKA has phosphorylated PDE4D and given our observation of PDE4D hyper phosphorylation in *CC2D1A* mutant cells, we tested if CC2D1A binds PDE4D thereby reducing phosphorylation and activation. The wt and *CC2D1A* mutant MEF cells were stimulated with forskolin for different lengths of time, then collected and lysed, protein concentrations were normalized and endogenous PDE4 activity assayed. While PDE4 activity increases and decreases gradually with increasing time of forskolin stimulation in wt cells, PDE4 activity is higher in *CC2D1A* mutant cells even before stimulation and increases rapidly after the first time point of forskolin stimulation and stays elevated for longer (Figure 2D) indicating that CC2D1A affects PDE4 activity. To test whether this regulation occurs as a result of CC2D1A-PDE4D binding, we first used the PDE4D5 (IX) plasmid and the GST- CC2D1A (I) plasmid (Figure 1) to assay PDE4D5 recombinant activity before and after *in vitro* phosphorylation by PKA and found that PDE4D5 activity increases approximately two fold after phosphorylation by PKA (Figure 2E) and this is consistent with the previously published data [21,22]. Then the effect of CC2D1A-PDE4D binding on PDE4D5 activity *in vitro* was examined by incubating GST-CC2D1A (I) protein with PDE4D5 (IX) in the presence and absence of PKA. When CC2D1A was bound to PDE4D5 the activity was not affected by PKA suggesting that CC2D1A-binding PDE4D may prevent activation by PKA phosphorylation (Figure 2E). This is supported by the fact that PDE4D5 activity increased after incubation with PKA and prior to the addition of CC2D1A (I) (Figure 2E). To further investigate if this regulation acts by preventing the PDE4D phosphorylation by PKA, we incubated GST- CC2D1A (I) with PDE4D5 (IX) for *in vitro* binding, added PKA for *in vitro* phosphorylation and western blot to examine PDE4D5 phosphorylation at (S^126^). The results show that PDE4D5 phosphorylation is dramatically reduced after binding to full-length CC2D1A (I) (Figure 3A) while PDE4D5 phosphorylation increased after incubation with PKA and prior to the addition of CC2D1A (I) (Figure 3A and B). PDE4D5 (IX) activation by PKA was assayed after interaction with different CC2D1A fragments (Figure 1A) *in vivo* to determine which DM14 domains are critical for PDE4D activity. PDE4D5 (IX) displayed normal activity before and after activation by PKA (Figure 3C) and could be inhibited by the PKA inhibitor (PKI) indicating that the activity is a consequence of the activation by PKA (Figure 3C). In agreement with the PDE4D5 phosphorylation results (Figure 3A), PKA does not appear to effect PDE4D5 activity after pre-incubation with the full length CC2D1A (I) and CC2D1A (III and IV) fragments separately (Figure 3C). Although *in vitro* binding results confirm that the first DM14 domain (Figure 1C) is essential for CC2D1A-PDE4D binding, the results from (Figure 3C) suggest that fragment VI, cannot prevent the increase in PDE4D5 activity after PKA-dependent phosphorylation. The results therefore suggest that the first three DM14 domains (fragment IV) are required to substantially reduce of PDE4D5 activity. Based on that, we conclude that the first three DM14 domains are required to reach near wt regulation of PDE4D5 activity (Figure 3C). The CC2D1A-C2 (VII) fragment does not prevent the increase in PDE4D5 activity after PKA-dependent phosphorylation (Figure 3C).

### Rolipram, a PDE4 specific inhibitor restores the CREB S133 (S^133^) phosphorylation in CC2D1A mutant cells

**Figure 4:**
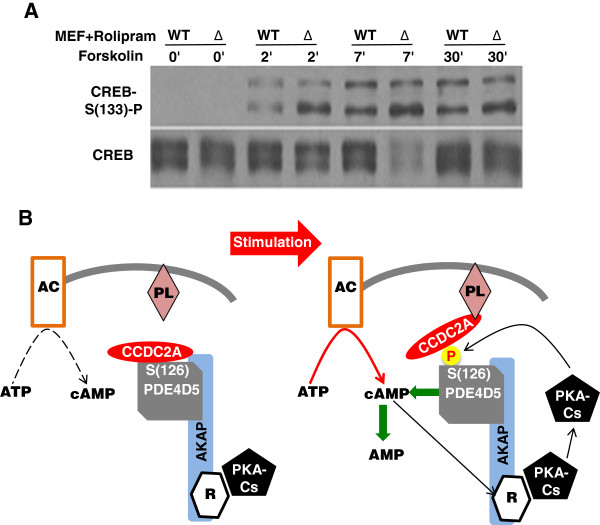
**Model of CC2D1A-PDE4D interactions. A**. Western blot of time course forskolin induction (0, 2, 7 and 30 minutes) of wt and CC2D1A mutant (Δ) MEF cells after pre incubation with Rolipram, a specific PDE4 inhibitor, probed with anti-phospho CREB (S^133^) or anti-CREB. **B**. Schematic model of the mechanism by which CC2D1A regulates the negative feedback mediated by PDE4D.

Since PDE4 is more active in CC2D1A mutant cells, we hypothesized that suppressing PDE4 activity may compensate for the defective phosphorylation of the PKA target CREB at (S^133^) in *CC2D1A* mutant cells [17]. To test this we treated wt and *CC2D1A* mutant MEF cells with a PDE4 specific inhibitor Rolipram [23] prior to stimulation with forskolin, monitored subsequent CREB phosphorylation at (S^133^). Intriguingly, the results indicated that CREB (S^133^) phosphorylation in the *CC2D1A* mutant cells was restored to wt levels suggesting that PDE4-hyper activity in the mutant might be lowering the cAMP levels leading to defective PKA activity and thereby defective CREB (S^133^) phosphorylation (Figure 4A).

The cAMP-dependent signaling is essential for many cellular processes including cellular homeostasis and development [24,25]. Consequently, spatial and temporal regulation of cellular cAMP concentrations needs to be maintained under tight control. This control is largely exerted by PDEs [26] and more recently, CC2D1A has also been implicated in the control of cAMP homeostasis [17]. The CC2D1A protein contains four DM14 domains (Figure 1A) that in turn are annotated only on the basis of amino acid sequence comparisons, but their biochemical and cellular functions have remained elusive [20,27].

While essential and sufficient for binding, the first DM14 of CC2D1A is not sufficient to confer wt function. Humans lacking the fourth DM14 domain of CC2D1A are intellectually disabled but have no other discernible symptoms suggesting that CC2D1A-dependent regulation is particularly critical in developing neural tissue. In turn, the mouse with a CC2D1A mutation that lacks all but the first DM14 has an even more severe phenotype causing death shortly after birth [17]. The results suggest that the absence of DM14 domains 2 and 3 leads to PDE4D hyper-phosphorylation at (S^126^), a reaction that is catalyzed by PKA. This hyper-phosphorylation leaves PDE4D constitutively activated and consequently disturbs cAMP homeostasis and cAMP-dependent downstream processes and notably CREB phosphorylation at (S^133^). The latter can be restored by the PDE4 inhibitor Rolipram suggesting that suppressing PDE4D activity may alleviate the effects of the defective phosphorylation of the PKA target CREB at (S^133^) in *CC2D1A* mutant cells. If CREB phosphorylation is disturbed, it is likely to lead to neural defects and abnormal brain development causing impairments in mental function [10]. The fact that Rolipram has therapeutic benefits as an antidepressant and as an antipsychoticum [28] is further indirect evidence that PDE4D may play a key role in the nervous system and it is noteworthy that disturbances in intracellular cAMP levels and PKA-dependent CREB phosphorylation have recently been reported to cause defects in neural crest lineages which in turn manifest themselves as Familial Dysautonomia (FD) syndrome [29]. Given that the mutant CC2D1A protein in NSID patients has the first three DM14 domains intact but is lacking the fourth, we believe that the fourth domain also has a role in CC2D1A regulating PDE4D5 and may be causative for the human syndrome. We speculate that CC2D1A binding to phospholipids [17] at the membrane introduces conformational changes exposing the PDE4D5 (S126) allowing its phosphorylation and activation. Indeed, our ongoing research indicates that the fourth DM14 domain assures the correct *in vivo* CC2D1A configuration prior to binding to the phospholipid. If this configuration is impaired it is likely to affect PDE4D5 regulation *in vivo*, and with it cellular cAMP homeostasis. However, the biological role and molecular mechanism of the fourth DM14 domain awaits further testing *in vivo*.

Here we propose a model (Figure 4B) that links spatial observations to structural and functional aspects of cAMP-dependent phosphorylation. Spatial association of CC2D1A with PDE4D both in the cytosol and, after cAMP stimulation, at the periphery suggests that the common localization may be part of cAMP homeostasis and the regulation of cAMP-dependent processes. In the proposed model for PDE4D5 regulation, upon activation of the adenylate cyclase, cAMP levels increase and cAMP-dependent signaling occurs. The CC2D1A–PDE4D–PKA complex relocates to the plasma membrane along a cAMP gradient where CC2D1A will prevent the early PDE4D phosphorylation and activation by PKA. At the membrane, CC2D1A anchors the complex to the cell membrane by binding phospholipids and modulate PKA activity by keeping PDE4D5 inactive for longer allowing a longer signal duration (Figure 4B). Binding of the C2 domain of the CC2D1A to a membrane phospholipid [17] might cause conformational changes in CC2D1A exposing the (S^126^) residue of PDE4D5 that in turn will be activated by the catalytic subunit of PKA that is released after cAMP activation (Figure 4B). Binding of CC2D1A to the phospholipid un-protects (S^126^), allowing PDE4D5 activation that then reduces cAMP levels and turns the signal “off”. We propose that CC2D1A regulates the cAMP/PKA pathway through fine-tuning the negative feedback loop that acts via PKA activation of PDE4D. When this pathway is defective, e.g. during neuronal development, it may lead to a state of compromised dendrite growth, synapse formation and neuronal survival [17] and this in turn may be a cause of NSID.

Given that constitutive PDE4D phosphorylation at the PKA site leads to higher enzymatic activity in *CC2D1A* mutant cells, we propose that *CC2D1A* has a role in preventing early PDE4D (hyper-) phosphorylation and activation which in turn would enable higher amplitude of cAMP and longer signal duration to exert the full function effects. Our finding revealed the uncharacterized function of the DM14 domain that, if proven *in vivo*, may be used in the future as a target for novel therapeutic agents in treatment of diseases caused by PDE4D5-linked disorders.

## Conclusion

The findings presented here suggest that CC2D1A with its DM14 domains is a novel regulator of PDE4D and that specific and spatially correct binding of CC2D1A to PDE4D regulates the activity of the phosphodiesterase that is necessary for fine-tuning cAMP-dependent downstream signaling. We speculate that CC2D1A may be a promising target for therapeutic interventions in conditions with impaired PDE4D5 function such as NSID.

## Material and methods

### Glutathione S-transferase (GST)-CC2D1A fusion protein

A BamHI-EcoRI cDNA fragments encoding the proteins and protein fragments (see Figure 1) cloned into the vector pGEX-4T1 using CC2D1A cDNA in a V5 vector as template. The PCR product and pGEX-4T were digested with BamHI and EcoRI, gel purified, and ligated over night at 16°C. Ligated plasmids were transformed into *E*. *coli* DH5α and plated on agarose gel plates with Ampicillin for selection. For GST-fusion protein purification, overnight cultures of the pGEX-CC2D1A strains were grown in Luria-Bertani broth (LB) with 50 μg/ml of Ampicillin to an optical density of 0.45 at 600 nm. Isopropyl β-D-1-thiogalactopyranoside (IPTG) was added to a final concentration of 0.4 mM, and the mixture was placed in a shaker at 37°C for 4 h. Cells were pelleted and resuspended in 5 ml of phosphate-buffered saline (PBS) plus protease inhibitors (0.5 mM phenylmethylsulfonyl fluoride, 0.8 mg/ml leupeptin, 0.8 mg/ml pepstatin, and 0.1 mM EDTA) and the mixture was left on ice for 10 min. and then sonicated three times for 10 seconds each. Triton X-100 was then added to a final concentration of 0.1%, and the lysate was shaken gently at 4°C for 30 min. Cell debris was cleared by centrifugation at 15,000 rpm at 4°C for 30 min., and the supernatant was transferred to a new microcentrifuge tube with 100 μl of 5% slurry glutathione-conjugated beads. The lysate-GST bead slurry was incubated for 1 h at 4°C with gentle rocking. After that, the slurry was centrifuged at 1,000 rpm for 30 s, the supernatant was removed, and the beads were gently resuspended in 500 μl PBS. The centrifugation-resuspension was repeated three times to wash the beads free of most contaminating lysate proteins. The GST beads were then resuspended in 100 μl of PBS for further use. To cleave the GST tag from the proteins biotinylated Thrombin (Novagen) was used. First, 10 μl of thrombin was added (1 unit/μl in 50 mM sodium citrate, pH 6.5, 200 mM NaCl, 0.1% Polyethylene glycol (PEG)-8000, 50% glycerol) to the 200 μl of the bead slurry (100 μl beads and 100 μl PBS), followed by 20 μl of 10X thrombin cleavage buffer (200 mM Tris–HCl pH 8.4, 1.5 M NaCl, 25 mM CaCl2) and the mixture was gently shaken at room temperature for 8 h. To capture the thrombin, 200 μl of streptavidin agarose beads (50% slurry, Novagen) were added to each sample and incubated with gently shaking at room temperature for 30 min. After that, samples were centrifuged at 500 × g for 3 min. and the supernatant containing the cleaved protein was transferred to a new tube.

### Analysis of signaling pathways in cultured cells

Cells were stimulated with Forskolin (Sigma) for 5, 10, 15 and 30 min., washed in cold PBS and collected. For inhibition of PDE4 within cells, wild-type and CC2D1A mutant MEF cells were treated with 10 μM Rolipram for 20 min. then stimulated with 20 μM forskolin for 2, 7, and 30 min. Whole cell lysates (15 μg of protein) were separated by SDS-PAGE gel and transferred to nitrocellulose membranes. The membranes were incubated for 1 h at room temperature, or overnight at 4°C, in the corresponding primary and secondary antibodies membranes developed using the enhanced chemiluminescence (ECL) detection system (Amersham Biosciences).

### Immunocytochemistry

Cells were cultured on poly-D-lysine-coated cover slips and after experimental treatments the cells were fixed with 4% paraformaldehyde in PBS for 10 min. at room temperature, washed 3 times with PBS, incubated for 20 min. at 37°C in PBS containing 5% goat serum, prior to adding the Anti-PDE4D and Anti-CC2D1A primary antibodies. Following three washes with PBS, the coverslips were incubated for 20 min. at 37°C with the Texas Red and Fluorescein isothiocyanate (FITC) conjugated secondary antibody (1/300 dilution, Chemicon). After three washes with PBS the cover slips were incubated for 5 min. on ice in PBS containing 50 μg/ml of 4′,6-diamidino-2-phenylindole (DAPI), then washed three times with PBS and mounted on glass slides. Antibody signals were visualized with a Delta Vision de-convolution microscope.

### In vitro protein binding assay

We obtained a GST-PDE4D5 plasmid from Graeme B. Bolger (University of Alabama Comprehensive Cancer Center). GST-PDE4D5 (VIII) protein was expressed and purified and cleaved as described above. The four GST-tagged CC2D1A (I, II, III, and IV) proteins that were previously purified on GST beads, and the purified PDE4D5 (IX) protein, were used as follows. The PDE4D5 (IX) (20 μg) was added to the 200 μl of the GST bead solution with bound GST-CC2D1A (I), GST-CC2D1A (II), GST-CC2D1A (III), or GST-CC2D1A (IV) separately. After incubation at 4°C for 4h, samples were centrifuged at 500 x g for 1 min. and the supernatant was removed. Washing with PBS and centrifugation were repeated three times and the PBS from the last wash was totally removed. Samples were boiled at 95°C with 30 μl of protein loading buffer for 5 min. To assess binding, 20 μl of each sample was loaded on an SDS-PAGE gel and duplicate western blots were made and stained with anti-PDE4D affinity-purified rabbit antibody (1/500; FabGennix), or anti-GST mouse monoclonal antibody (GenScript) separately.

### PDE4 Assay

The wt and CC2D1A mutant Mouse Embryonic Fibroblasts (MEF) cells were stimulated with Forskolin, processed and the broken up by sonication. Phosphorylation of the GST-PDE4D5 recombinant protein was assessed after PKA treatment and exposure to ATP (Assay Designs). PDE4 activity was measured using the PDE4 Enzymatic Assay Kit (FabGennix) according to the manufacture’s protocol. (The methods applied here are further detailed in the Additional file 1).

## Abbreviations

ACs: Adenylate cyclases; AKAP: A kinase anchoring protein; cAMP: Cyclic adenosine 3^′^,5^′^-monophosphate; CC2D1A: Coiled-coil and C2 domain-containing 1A; CREB: cAMP response element-binding protein; DM14: *Drosophila melanogaster* 14 protein; GST: Glutathione S-transferase; MEF: Mouse embryonic fibroblasts; NSID: Non syndromic intellectual disability; PDE: Phosphodiesterase; PKA: cAMP-dependent protein kinase A.

## Competing interests

Both authors declare that they have no competing interests.

## Authors’ contributions

AA has designed and performed the experiments; AA and CG have interpreted the data, conceived the model and written the manuscript. Both authors read and approved the final manuscript.

## Supplementary Material

Additional file 1 Materials and Methods. Click here for file
