# Supplementary material for: Phosphodiesterase activity is regulated by CC2D1A that is implicated in non-syndromic intellectual disability
Source: Cell Commun Signal. 2013 Jul 4;11:47. doi: 10.1186/1478-811X-11-47 (PMC3704924; doi:10.1186/1478-811X-11-47)
Supplement: Additional file 1 — Materials and Methods. [file 1478-811X-11-47-S1.docx]

**Supplementary “Material and Methods”**

**Glutathione S-transferase (GST)-CC2D1A fusion protein constructs**

A BamHI-EcoRI cDNA fragment encoding the CC2D1A full-length, the N-terminal 620 amino acids of CC2D1A, the C-terminal 320 amino acids of CC2D1A, the first DM14 domain of CC2D1A only, the first two DM14 domains of CC2D1A, the first threeDM14 domains of CC2D1A and a CC2D1A deletion of the N-terminal 750 amino acids (missing the first DM14 domain) were inserted into the BamHI/EcoRI site of the vector pGEX-4T1 (GE Healthcare Life Sciences). By using a forward and reverse primer, polymerase chain reaction (PCR) was performed with CC2D1A cDNA in a V5 vector as template. The PCR product and pGEX-4T were digested with BamHI and EcoRI, gel purified, and ligated over night at 16°C. Ligated plasmids were transformed into *E. coli* DH5α and plated on agarose gel plates with Ampicillin for selection.

**GST-fusion protein purification.**

Overnight cultures of the pGEX-CC2D1A strains were grown in Luria-Bertani broth (LB) with 50 µg/ml of Ampicillin. A total of 100 ml of LB with Ampicillin was inoculated with 3 ml of the overnight cultures and grown to an optical density of 0.45 at 600 nm. Isopropyl β-D-1-thiogalactopyranoside (IPTG) was added to a final concentration of 0.4 mM, and the mixture was placed in a shaker at 37°C for 4 h. Induced cells were pelleted and resuspended in 5 ml of phosphate-buffered saline (PBS) plus protease inhibitors (0.5 mM phenylmethylsulfonyl fluoride, 0.8 mg/ml leupeptin, 0.8 mg/ml pepstatin, and 0.1 mM EDTA) and the mixture was left on ice for 10 min. and then sonicated three times for 10 seconds each. Triton X-100 was then added to a final concentration of 0.1%, and the lysate was shaken gently at 4°C for 30 min. Cell debris was cleared by centrifugation at 15,000 rpm at 4°C for 30 min., and the supernatant was transferred to a new microcentrifuge tube with 100 µl of 5% slurry glutathione-conjugated beads. The lysate-GST bead slurry was incubated for 1 h at 4°C with gentle rocking. After that, the slurry was centrifuged at 1,000 rpm for 30 s, the supernatant was removed, and the beads were gently resuspended in 500 µl PBS. The centrifugation-resuspension was repeated three times to wash the beads free of most contaminating lysate proteins. The GST beads were then resuspended in 100 µl of PBS and 5% of the beads were used for quantification of protein on Coomassie-stained sodium dodecyl sulfate polyacrylamide gel electrophoresis (SDS-PAGE) gels. To cleave the GST tag from the proteins biotinylated Thrombin (Novagen) was used. First, 10 µl of thrombin was added (1 unit/µl in 50 mM sodium citrate, pH 6.5, 200 mM NaCl, 0.1% Polyethylene glycol (PEG)-8000, 50% glycerol) to the 200 µl of the bead slurry (100 µl beads and 100 µl PBS), followed by 20 µl of 10X thrombin cleavage buffer (200 mM Tris-HCl pH 8.4, 1.5 M NaCl, 25 mM CaCl2) and the mixture was gently shaken at room temperature for 8 h. To capture the thrombin, 200 µl of streptavidin agarose beads (50% slurry, Novagen) were added to each sample and incubated with gently shaking at room temperature for 30 min. After that, samples were centrifuged at 500 x g for 3 min. and the supernatant containing the cleaved protein was transferred to a new tube. The extent of cleavage of the samples was determined by SDS-PAGE comparison of the recovered supernatant and the bead pellet.

Primers sequences:

5' GST-BamHI-CC2D1A (N1): 5'-GCG GAT CCA TGC ACA AGA GGA AAG GA-3'

3'GST-EcoRI-CC2D1A (N2): 5'-GCG AAT TCC AGC TGG GTG AAT TGG TT-3'

3'GST-EcoRI-CC2D1A (N3): 5'-TTG GTC CCT AGG GTC CCG CTT AAG-3'

3'GST-EcoRI-CC2D1A (N4): 5'-GCG GAT CCC ACC TGT GGA ATG GAA GC-3'

3'GST-EcoRI-CC2D1A (N5): 5'-GTC TTC CCG TCC AGA TAT CTT AAG CG-3'

3'GST-EcoRI-CC2D1A (N6): 5'-GCG AAT TCC AGC TGG GTG AAT TGG TT-3'

3'GST-EcoRI-CC2D1A (N7): 5'-TCG AAC CAG ACG ACC AAC CTT AAG CG-3'

5' GST-BamHI-CC2D1A (C): 5'-GCG GAT CCG GCA ACA TCA CTG AAA CC-3'

3'GST-EcoRI- CC2D1A (C): 5'-GCG AAT TCT CAC CTG CGG AGC CGC TG-3'

**Producing MEF cells**

Procedure was performed in cell culture hood. Freshly harvested Wild type and Mutant letter mates embryos (18 days) were placed in 10cm cell culture dish separately, and covered in PBSA and remove placental and other maternal tissues. The head was cut away and used for DNA isolation for genotyping. After that embryos were eviscerated (removing all innards). Then, embryos bodies were placed in separate new clean 10cm plate. 1mL trypsin was added to each plate and embryos were minced with sterilized razor blade. Plates were placed in 37°C incubator for 45min (tilted so trypsin covers the cells). Trypsin activity was quenched by adding 4mL MEF media (DME w/ 10% FCS+P/S) to each plate and Pipetted 10-20x to break up tissues. Cellular suspension was transferred to T75 flask and 15mL MEF media was added. Cells were allowed to grow to confluence (3-4days) then medium was aspirated off and rinse with 10mL PBS. 3mL trypsin were added to each flask and incubate in 37°C incubator 5-10min. Trypsin activity was quenched by adding 7mL MEF media and Pipetted 15x to resuspended cells. Suspension was transferred to two new T175 flasks and 50mL MEF media was added to each flask. Cells were allowed to grow to confluence (3-4 days) then harvested via trypsin and frozen down.

**Analysis of signaling pathways in cultured cells**

Cells were stimulated with Forskolin (Sigma) for 5, 10, 15 and 30 min., washed in cold PBS and collected by scraping from culture plates into 2.5 cell culture volumes of NETN lysis buffer (150 mM NaCl, 1 mM EDTA, 50 mM Tris-HCl (pH 7.8), 1% Nonidet P-40, 1 mM phenylmethylsulfonyl fluoride, 0.5 µg/ml leupeptin, and 0.5 µg/ml pepstatin). For inhibition of PDEs within cells, wild-type and CC2D1A mutant primary macrophage cells were treated with 10 µM Rolipram for 20 min. then stimulated with 20 µM Forskolin for 2, 7, and 30 min. Afterwards, cells were washed with PBS, collected and incubated in NETN, plus a protease inhibitor cocktail, on ice for 15 min. followed by sonication. The cells were broken by sonication and cell debris was removed by centrifugation at 13,000 x g for 20 min. (4°C). Protein concentrations were determined with the Bio-Rad reagent. Whole cell lysates (15 µg of protein) were separated by SDS-PAGE gel and transferred to nitrocellulose membranes. The membranes were incubated for 1 h at room temperature, or overnight at 4°C, in the corresponding primary antibody [anti-phospho-CREB monoclonal antibody (1/1000 dilution, Cell Signaling) in 5% bovine serum albumin (BSA) in Tris-buffered saline with Tween(TBST) ( 500 ml 0.1% Tween-20 in 1x Tris- buffered saline - pH 7.4), anti- CC2D1A polyclonal antibody (1/5000 dilution, Bethyl) in 5% milk in TBST, anti-total CREB monoclonal antibody in 5% BSA in TBST (1/1000 dilution, Cell Signaling), anti-actin monoclonal antibody in 5% milk in TBST (1/8000 dilution, Sigma), anti phospho-PDE4D affinity purified rabbit antibody (1/500, FabGennix) in 5% BSA in TBST overnight at 4°C, and anti PDE4D affinity-purified rabbit antibody (1/500) in 5% BSA in TBST (FabGennix)]. Following the incubations with primary antibodies, the membranes were washed three times with TBST for 15 min. each time. Afterwards, the membrane was incubated for 1 h in the secondary antibodies [horseradish peroxidase-linked anti-mouse Immunoglobulin G (IgG) antibody in 5% milk in TBST (1/5000 dilution, Amersham Bioscience) or a horseradish peroxidase-linked anti-rabbit IgG antibody in 5% milk in TBST (1/8000 dilution, Amersham Life Science). In the end the membrane was developed using the enhanced chemiluminescence (ECL) detection system (Amersham Biosciences).

**Immunocytochemistry**

Glass 12 mm circular coverslips (Fisher) were heated in loosely covered glass beaker in 1M HCL at 55^o^C for 16 h, cooled and washed extensively with sterile deionized water. Coverslips were transfer to a 100 mm Petri dish, washed with 70% ethanol, put on a sheet of Whatman filter paper and left to dry. The coverslips were transferred into 10 mm Petri dishes, covered with filter-sterilized poly-D-lysine solution (1mg/ml, Sigma), covered, sealed with parafilm, and gently agitated on a shaker at room temperature for 45 min. Coverslips were washed 5 times with deionized water, rinsed in 100% ethanol, separated and dried on sterile Whatman filter paper under Ultraviolet (UV) light. Coverslips were transferred to a sterile dish, sealed with parafilm and stored in 4o for future use. Cells were cultured on poly-D-lysine-coated cover slips as described above. After experimental treatments the cells were fixed with 4% paraformaldehyde in PBS for 10 min. at room temperature, washed 3 times with PBS, incubated for 10 min. on ice with PBS containing 0.3% Triton X-100, and washed again 3 times with PBS. Thereafter, the cover slips were incubated for 20 min. at 37°C in PBS containing 5% goat serum, and then the corresponding primary antibodies were added and incubated for 20 min. at 37°C [anti-CC2D1A (1/500 dilution, Bethyl), or anti- PDE4D affinity-purified rabbit antibody (1/500) in 5% BSA in TBST (FabGennix)]. Following three washes with PBS, the coverslips were incubated for 20 min. at 37°C with the Texas Red or Fluorescein isothiocyanate (FITC) conjugated secondary antibody (1/300 dilution, Chemicon). After three washes with PBS the cover slips were incubated for 5 min. on ice in PBS containing 50 µg/ml of 4',6-diamidino-2-phenylindole (DAPI), then washed three times with PBS and mounted on glass slides. Antibody signals were visualized with a Delta Vision deconvolution microscope.

***In vitro* protein binding assay**

We obtained a GST-PDE4D5 plasmid from Graeme B. Bolger (University of Alabama Comprehensive Cancer Center). GST-PDE4D5 protein was expressed and purified as described above. The four GST-tagged CC2D1A proteins that were previously purified on GST beads, and the purified PDE4D5 protein, were used as follows. The cleaved and purified PDE4D (20 µg) was added to the 200 µl of the GST bead solution with bound GST-CC2D1A full-length, GST-CC2D1A∆N, GST-CC2D1A∆C, or GST-CC2D1A first DM14, GST-CC2D1A first two DM14 and GST-CC2D1A first three DM14 separately. After incubation at 4°C for 4h, samples were centrifuged at 500 x g for 1 min. and the supernatant was removed. Washing with PBS and centrifugation were repeated three times and the PBS from the last wash was totally removed. Samples were boiled at 95°C with 30 µl of protein loading buffer for 5 min. To assess binding, 20 µl of each sample was loaded on an SDS-PAGE gel and duplicate western blots were made and stained with anti-PDE4D affinity-purified rabbit antibody (1/500; FabGennix), or anti-GST mouse monoclonal antibody (GenScript) separately.

**PDE4 Assay**

Wild type and CC2D1A mutant Mouse Embryonic Fibroblasts (MEF) cells were stimulated with Forskolin for 0, 7, 15 and 30 min. Afterwards, cells were washed with PBS, collected and incubated in NETN, plus a protease inhibitor cocktail, on ice for 15 min. followed by sonication. The cells were broken by sonication and cell debris was removed by centrifugation at 13,000 x g for 20 min. (4°C). Protein concentrations were determined with the Bio-Rad reagent. For the enzyme phosphorylation, the GST-PDE4D5 recombinant protein was incubated for 20 min. with PKA purified enzyme and Adenosine-5'-triphosphate (ATP) (Assay Designs). Whole cell lysates or GST fusion proteins (25 µg of protein) from each sample were used for the PDE4 assay. PDE4 activity was measured using the PDE4 Enzymatic Assay Kit (FabGennix) according to the manufacture’s protocol. Briefly, duplicate rows of 1.5 ml plastic conical tubes with snap lock caps were placed on ice. Twenty five µl of whole cell lysates or GST fusion proteins were added to one set of the duplicate rows on ice. Conical tube placed on ice at 4°C. 25 µl of Rolipram (100 µM) were added to one set of the duplicate rows on ice. 25µl of incubation buffer were added to the set of tubes without Rolipram conical tubes placed on ice at 4^o^C to normalize the samples volume. 50µl of buffered PDE substrate [PDE substrate in substrate buffer and [2,8-3H]cAMP ammonium salt in ethanol [25-40 curie/ millimole (Ci/mmole) prepared fresh] were pipetted directly in to all of the conical tubes and incubate for 10 min. at 30°C with shaking. After 10 min. at 30°C, all tubes were immediately transferred to a 100°C water bath for 3 min. to stop the PDE reaction. After 3 min. in the water bath all tubes were immediately transferred on ice to chill the reaction. For the conversion of AMP in to adenosine, a 25 µl of the second enzyme solution were added to each tube and incubated at 30°C for 15 min. of the second enzymatic reaction to equilibrium. Four hundred µl of the pre-activated ion exchange resin were added to each tube, caped and vortexed for 30 s. Incubate all tubes on ice for 15 min. Then all tubes were centrifuged at 13,000 rpm for 2 min. and 150 µl of supernatant were carefully transferred for 3H beta liquid scintillation counting. Scintillation vials were kept at room temperature for at least 2 hours before counting. The PDE4 activity was calculated by subtracting non-PDE4 activity from Total PDE activity. The specific activity for PDE4 is defined as micromoles (µM) of cAMP hydrolyzed/minute/mg protein.
